# Interprofessional education initiatives to teach advocacy in health professions programs: a narrative review

**DOI:** 10.1007/s10459-026-10502-6

**Published:** 2026-01-22

**Authors:** Jessica Lees, Flynn Halliwell, Melissa Russell, Louisa Ng, Jayne Lysk, Fiona Dobson

**Affiliations:** 1 Department of Physiotherapy, School of Health Sciences, The University of Melbourne, Melbourne, Australia; 2 Melbourne Medical School, The University of Melbourne, Melbourne, Australia; 3 Melbourne School of Population and Global Health, The University of Melbourne, Melbourne, Australia; 4 Centre of Epidemiology and Biostatistics, Melbourne School of Population and Global Health, The University of Melbourne, Melbourne, Australia; 5 Department of Medical Education, The University of Melbourne, Melbourne, Australia

**Keywords:** Interprofessional education (IPE), Advocacy, Health professions education (HPE)

## Abstract

Interprofessional education (IPE) offers a valuable platform for advocacy education in health professions programs, reflecting the collaborative principles central to advocacy practice. This narrative review aimed to interpret and synthesise how advocacy is conceptualised, delivered, and experienced in IPE initiatives within health professions education (HPE). A narrative review approach was used to synthesise empirical literature to provide a comprehensive understanding of teaching advocacy in interprofessional HPE contexts. Five databases were searched using terms related to IPE and advocacy. Titles and abstracts were screened on predetermined criteria, and a consensus process was used to include studies following full text review. Thematic analysis was used to explore how the initiatives were structured, delivered, and experienced by learners. The search identified 941 studies, with nine meeting the inclusion criteria. While diverse in content, delivery mode, and duration, all initiatives incorporated a scaffolded pedagogical design. Teaching typically progressed from foundational knowledge-building to applied advocacy activities and reflection. Community involvement was a key feature in most initiatives. Students generally perceived the programs as relevant and impactful, reporting gains in advocacy knowledge and skills, as well as increased motivation to engage in advocacy in the future. A scaffolded interprofessional education curriculum that includes engagement with community is a valuable approach to teach health professions students about advocacy. Further research is needed to explore longer-term outcomes and the influence of interprofessional processes on advocacy learning.

**Supplementary Information:**

The online version contains supplementary material available at https://doi.org/10.1007/s10459-026-10502-6.

## Introduction

Advocacy across the health professions refers to the process of advancing individual, community and population health through both enabling access and navigation of healthcare and health systems, as well as taking action to address the upstream determinants of health. In their seminal paper on health advocacy, Hubinette et al. (2017) define health advocacy as “activities related to ensuring access to care, navigating the system, mobilizing resources, addressing health inequities, influencing health policy and creating system change” (Hubinette et al., 2017). Advocacy is a widely recognised responsibility of health professionals (Earnest et al., 2010; Kelland et al., 2014; Committee on the Robert Wood Johnson Foundation Initiative on the Future of Nursing atIoM, 2011; Kalaitzidis & Jewell, 2015), and is therefore a core graduate competency in many health professions education accreditation standards worldwide. While health professionals have often been integral agents of change in promoting health (Gaede & Pillay, 2019), it is important to acknowledge that at times, health professionals have also been complicit in actions that have caused harm or contributed to the marginalization and oppression of communities. Nevertheless, they remain persuasive and valuable contributors to contemporary advocacy efforts (Farrer et al., 2015). Farrer et al. (2015) highlight that training of health professionals in advocacy is an important method in overcoming systemic barriers to health equity, including discourses that position responsibility for health on the individual, rather than the structures which create and perpetuate ill-health (Farrer et al., 2015). There is also increasing discussion within the literature regarding the role for health advocacy within transformative learning approaches to health professions education, particularly regarding social accountability, community-based education and interprofessional education (Gaede & Pillay, 2019).

Despite the recognised importance of advocacy in the role of health professionals, significant inconsistencies and challenges have been identified in teaching and evaluating advocacy in health professions programs (Gaede & Pillay, 2019; McDonald et al., 2019; Dworkis et al., 2010; Blenner et al., 2017; Dharamsi et al., 2011; Brender et al., 2021; Douglas et al., 2018; Peltzer et al., 2016; Bessette et al., 2020; Hatchett et al., 2015; To & Sharma, 2015; Verma et al., 2005). Indeed, many health professions students express strong interest in advocacy and self-identify as advocates (Brender et al., 2021; Press et al., 2015), evidenced by a rich history of student involvement in health advocacy efforts (Schreidah et al., 2023). However, student involvement in advocacy often occurs with minimal educational support or guidance (Schreidah et al., 2023), with many students reporting low confidence in their knowledge and practical skill in health advocacy (Brender et al., 2021; McGrew et al., 2015). Advocacy in health professions education is closely tied to understanding and addressing the social determinants of health, which shape health outcomes and access to care. Whilst the inclusion of social determinants of health is an important and increasingly common foundation in health professions curriculum (Hubinette et al., 2017), Sharma et al. (2018) suggest that education must move beyond awareness of social determinants of health, and toward critical consciousness and active commitment to advancing social justice and health equity. Importantly, this requires structural and cultural transformation within HPE curricula to include advocacy education which is skills-based, action-oriented, interprofessional, and partnership-focused (McDonald et al., 2019; To & Sharma, 2015; Verma et al., 2005; Hubinette et al., 2015).

Interprofessional education (IPE) is uniquely positioned to teach advocacy to students across the health professions in a way which reflects the collaborative and transdisciplinary skills central to the practice of health advocacy itself. IPE involves learners from two or more health professions learning with, from and about each other in order to improve collaborative practice and quality of care (Barr, 2005). It is suggested that IPE ultimately has the capacity to result in downstream effects such as optimization of health services, strengthening of health systems, and improvement of health outcomes (Organization, 2010). A review by Guraya and Barr (Guraya & Barr, 2018) offers evidence that IPE can contribute meaningfully to the development of health professions students, with reported improvements in attitudes, knowledge and skills of health professions students. Their synthesis highlights the perceived value of IPE in shaping learners’ professional growth and collaborative readiness. Given the importance of collaborative interdisciplinary partnerships within the practice of health advocacy (Hubinette et al., 2014), it has therefore been suggested that IPE may offer a promising platform for teaching advocacy to health professions students (To & Sharma, 2015).

Despite the emergence of interprofessional advocacy education initiatives in health professions programs, a comprehensive synthesis of these efforts is currently lacking in the literature. This narrative review aimed to explore how advocacy education is conceptualised, delivered, and experienced within interprofessional health professions education curricula.

This review adopts the conceptualisation of advocacy put forward by Hubinette et al. (2017), which defines and delineates advocacy types and approaches. Within this framework, Hubinette et al. (2017) draw on Dobson et al. (2012), and their distinction between two *types* of advocacy, namely: ‘agency’, activities which support individuals to navigate the healthcare system; and ‘activism’, activities which challenge the system in order to influence the health of communities or populations at large. In their framework, Hubinette et al. (2017) also differentiate two *approaches* to advocacy: ‘directed’, where health needs are identified by health professionals and involves subsequent representation, prescription or guidance on an issue; and ‘shared’, which responds to community-identified needs, strengths and opportunities through supportive and collaborative partnerships in order to work with, rather than on behalf of, the individual, community or population. This conceptual framework informs the focus of this narrative review by providing an organizing lens for identifying and synthesizing the components and outcomes of interprofessional advocacy education initiatives in health professions education programs.

## Methods

We adopted a narrative review methodology to synthesise a diverse body of literature relating to interprofessional advocacy education in health professions programs. Narrative reviews allow for the inclusion of heterogeneous studies, methods, and perspectives, making them well suited to research questions that are interpretive and exploratory in nature. Narrative review methodology enables researchers to critically explore how concepts are constructed, enacted, and understood across diverse contexts. Rather than aggregate findings, this approach seeks to interpret, illuminate, and critically engage with the literature through a lens that values contextual nuance (Greenhalgh et al., 2018). This approach aligned with our conceptual framing, which foregrounds the socially situated, relational, and practice-based nature of advocacy education. It also enabled us to explore how advocacy is conceptualised, delivered, and experienced in interprofessional education settings. Our review was guided by the narrative synthesis process by Wong et al. (2013), and followed a staged process of literature search, data extraction, mapping of key elements, and interpretive synthesis.

The analysis was situated within a contextualist epistemology (Braun & Clarke, 2006), acknowledging both the reality of participants’ experiences and the interpretive role of the researcher in constructing meaning. The analysis drew on a reflexive thematic analysis approach, consistent with Braun et al. later work (2023), which emphasises researcher subjectivity, theoretical transparency, and analytic depth. This approach was selected to support a narrative review methodology aligned with interpretive paradigms, suitable for synthesising diverse forms of qualitative and mixed-methods data.

### Search strategy

An initial comprehensive search was conducted in MEDLINE, from which the search terms were refined (Appendix 1), and then the search was run across five databases (MEDLINE, EMBASE, PsychInfo, ERIC and CINAHL) in February 2024. The reference lists of included studies were systematically examined through hand-searching to identify additional relevant sources. Additionally, a grey literature search was performed using Google Scholar using the same search terms.

No date range restrictions were applied to either the database searches or the hand search using Google Scholar. This inclusive approach was chosen to allow for the identification of all relevant literature regardless of publication date. The included databases (MEDLINE, EMBASE, PsychInfo, ERIC, and CINAHL) each provide access to historical collections dating back to the mid-20th century (e.g., Ovid MEDLINE to 1946, ERIC to 1966 depending on indexing). Despite this wide temporal scope, the earliest study meeting our inclusion criteria was published in 2013, which likely reflects the relatively recent emergence of advocacy education as a focused area of interprofessional curriculum development.

### Inclusion/exclusion

The review focused on studies that explored educational programs involving students from two or more health professions, where advocacy was a central component of the curriculum initiatives. While this meets a broad definition of interprofessional education (IPE), we acknowledge that the included studies varied in the extent to which they actively engaged interprofessional *processes*, such as interprofessional communication, collaboration, and negotiating roles or professional boundaries. The decision to include studies involving only two health professions reflects the current limitations of the evidence base, rather than an ideal threshold for interprofessional learning. Studies were eligible if they reported outcomes related to advocacy education and were situated within a university-based health professions program. Only peer-reviewed studies published in English were included.

Studies were excluded if they described educational initiatives delivered as part of workplace training for post-graduation health professionals or if they were available only in abstract form (Table 1).

**Table 1:** Eligibility criteria

Inclusion
1. Participants in the educational program described in the study included students from two or more health professions;
2. Advocacy was a primary component of the education initiative;
3. Outcomes related to advocacy were evaluated and reported;
4. The education initiative occurred as part of a university program;
5. They were published in English language.

Exclusion
1. The education initiative occurred as part of workplace training for employed health professionals;
2. Available as abstract only.

Following duplicate removal, titles and abstracts were screened using Covidence software (Covidence, 2024). Full texts of studies were then retrieved and independently reviewed by two authors for final inclusion using a consensus process. Conflicts were resolved through discussion with a third author.

### Data collection

Information was gathered on the author(s) and year of publication, along with the country in which the study was conducted, to provide context regarding the geographical distribution and recency of the research (Appendix A). Study designs were noted to capture the range of methodological approaches employed across the included studies.

Participant data were extracted to provide insight into the breadth and diversity of representation. This included the total number of participants, the number of health disciplines represented, and the types of health disciplines involved. Details of the education initiative were also carefully documented, encompassing descriptions of the teaching methods used, frequency and duration of the initiative, and the other defining curricular features.

Data related to the impacts of each initiative were derived primarily through student-generated accounts, including written reflections, focus groups, and post-initiative survey responses. As such, the analysis was shaped by learners’ accounts of their experiences within the initiatives, particularly regarding what supported or constrained their engagement. While some studies included perspectives from educators or community partners, the dominant voice across the dataset was that of students. This interpretive emphasis informed the construction of Theme 3, which foregrounds student-reported enablers and challenges in advocacy learning.

Finally, the conclusions drawn by the authors were noted to provide insights into the perceived effectiveness and impact of the initiatives.

### Analysis

This research followed the narrative synthesis approach outlined by Wong et al. (2013). Hubinette’s conceptualisation of advocacy was used to interpret whether curriculum initiatives aligned with activism, agency or both, identify community involvement or collaboration, and discern if advocacy was the primary focus of the education initiative or one of multiple components.

The thematic analysis followed Braun and Clarke’s framework, involving an iterative and rigorous process of coding, theme development, and interpretative synthesis (Braun et al., 2023). The primary researchers inductively coded the studies’ components and outcomes. These coded items were then actively interpreted and organised into candidate themes and sub-themes through an iterative process by the two primary researchers. The two researchers then collaboratively refined and reviewed these themes, with input and oversight from the broader research team. This collaborative and reflexive process ensured that the final themes reflected a consensus grounded in the dataset, while also accommodating the diverse perspectives and disciplinary expertise of the team.

### Positionality of authors

The synthesis and thematic analysis were conducted primarily by JL and FH. JL is a physiotherapist with a PhD in Health Professions Education and experience designing and delivering advocacy curriculua. FH is a dual-degree student in postgraduate medicine and public health, with a strong interest in systems-level change and student agency in health education.

The broad research team includes academics and clinicians from medicine, physiotherapy, public health (epidemiology) and higher education, all of whom have been involved in the design of interprofessional advocacy education content within postgraduate health professions courses. This diversity of disciplinary perspectives contributed to a rich and multi-layered interpretation of the data.

As researchers, we acknowledge that our backgrounds, values, and professional commitments shaped the way we approached this review. Our shared orientation toward equity, social accountability, and collaborative practice sensitised us to how advocacy was framed and operationalised in the included studies. Throughout the analytic process, we engaged in reflexive dialogue and critical questioning, recognising that our interpretive lens may have influenced theme development and the prioritisation of certain findings. These reflexive practices were intentionally incorporated to enhance transparency and mitigate bias.

## Results

**Fig. 1:**
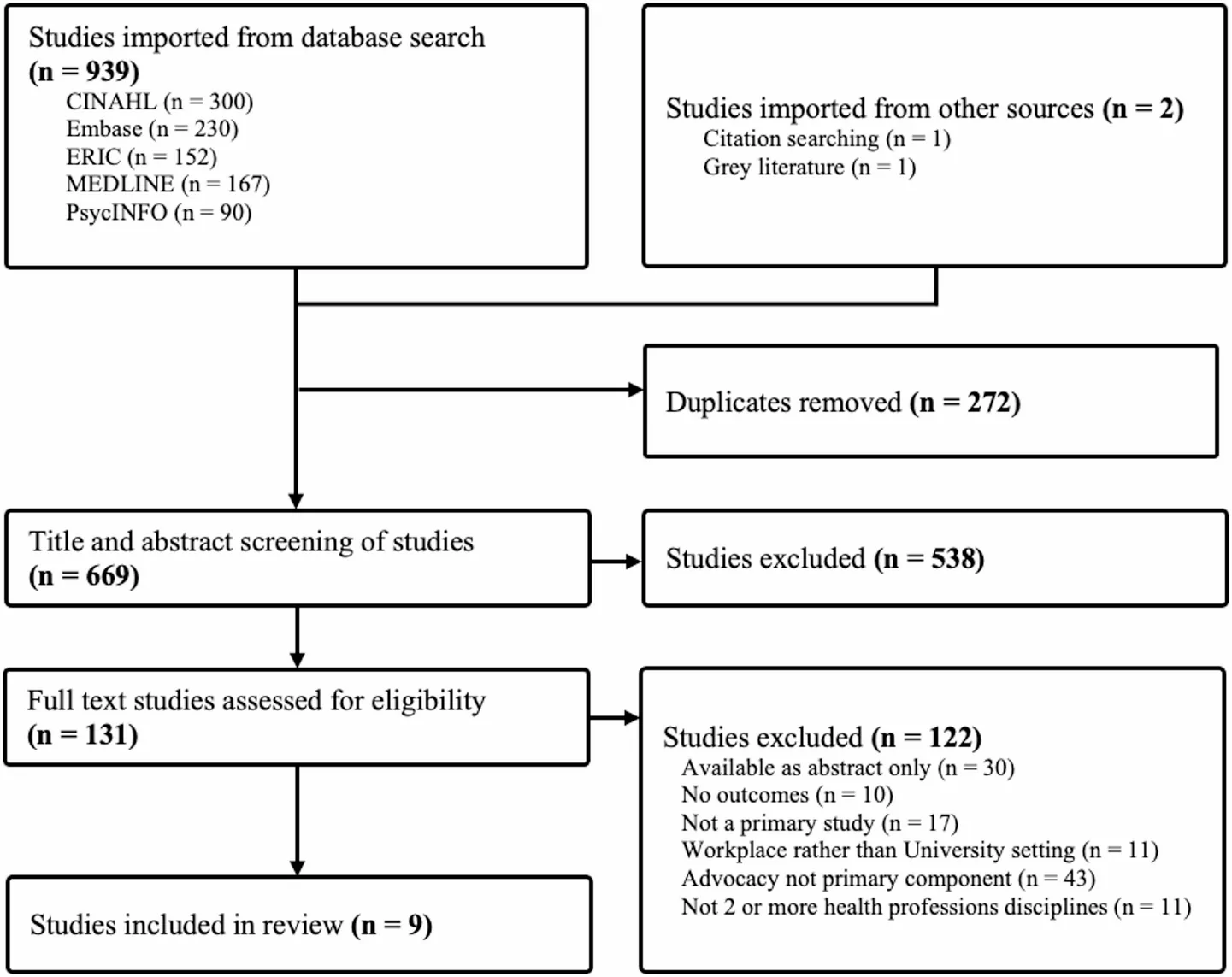
PRISMA diagram

The search identified 941 studies for screening, of which 272 were duplicates (Fig. 1). Following title and abstract screening of the 669 remaining studies, 131 studies were included for full text review, of which 122 studies were then excluded. The most common reason for exclusion was that advocacy was not a primary component of the educational initiative. While we did not conduct a systematic review, we utilized this structured nature of the PRISMA guidelines to guide reporting (Fig. 1) (Page et al., 2021). There were nine included studies (Adams et al., 2023; Eaton et al., 2017; Hallowell et al., 2024; Liller et al., 2020; Peluso et al., 2013; Yamaoka et al., 2019; Langlois & Lymer, 2016; Pinto et al., 2018; Machin & Jones, 2014). While we did not conduct a systematic review, we utilized this structured nature of the PRISMA guidelines to guide reporting (Fig. 1) (Page et al., 2021). There were nine included studies (Adams et al., 2023; Eaton et al., 2017; Hallowell et al., 2024; Liller et al., 2020; Peluso et al., 2013; Yamaoka et al., 2019; Langlois & Lymer, 2016; Pinto et al., 2018; Machin & Jones, 2014).

### Characteristics

The earliest study that met the inclusive criteria was conducted in 2013 (Peluso et al., 2013), and the latest was 2023 (Adams et al., 2023). Studies were located in the United States (*n* = 6) (Adams et al., 2023; Eaton et al., 2017; Hallowell et al., 2024; Liller et al., 2020; Peluso et al., 2013; Yamaoka et al., 2019), Canada (*n* = 2) (Langlois & Lymer, 2016, Pinto et al., 2018) and the United Kingdom (*n* = 1) (Machin & Jones, 2014) (Appendix 2). Study designs included one pre- and post evaluation (Eaton et al., 2017) and eight post curriculum evaluations (Adams et al., 2023; Hallowell et al., 2024; Liller et al., 2020; Peluso et al., 2013; Yamaoka et al., 2019; Langlois & Lymer, 2016; Pinto et al., 2018; Machin & Jones, 2014).

### Participants

There was notable variability in the number of participants within each study (range: 11–508). There was also variability in the number of health professions included (range: 2–8). Health disciplines represented across the studies included: medicine, nursing and midwifery, a broad range of allied health such as social work and physiotherapy. Two studies also included law students or health administration students alongside the health disciplines (Appendix 2).

### Thematic analysis

Three overarching themes were generated through thematic analysis of the initiatives’ reported components and outcomes. In this review, we constructed a set of linking themes that we interpreted through inductive analysis as patterns that transcended individual subthemes. These integrative themes allowed for a more nuanced interpretation of how advocacy education was conceptualised, delivered, and experienced across interprofessional contexts, reflecting a reflexive approach to thematic development. Theme 1 was “*Curriculum design”*, and the subsequent two themes both related to the outcomes of the curriculum. Theme 2 was “*Impact of advocacy curriculum on student engagement and growth”*, and Theme 3 was “*Student perspectives on advocacy curriculum: enablers*,* challenges”*.

### Theme 1: Curriculum design

This theme explores the educational design features that shaped how advocacy was taught across the reviewed interprofessional education (IPE) initiatives. Three interrelated sub-themes were constructed to represent the curricular logic observed across studies: (1.1) instructional approaches, (1.2) community involvement, and (1.3) advocacy focus. Together, these elements reflect how advocacy education was strategically embedded, or in some cases, constrained, within IPE contexts.

#### Instructional approaches

Across the nine studies, a variety of pedagogical strategies were employed to deliver advocacy content, including interactive small-group activities (e.g., debates, team-based learning), community site visits, advocacy projects, lectures, and written reflections (Table 2). While the duration of the education initiatives across the studies varied from 4 h (Eaton et al., 2017) to year-long (Pinto et al., 2018), every study employed multiple teaching methods, highlighting a recognition that advocacy education requires more than knowledge transmission. Instead, diverse pedagogical designs sought to engage students in thinking, doing, and reflecting as advocates.

A key interpretive insight drawn from the data was the prominence of a scaffolded instructional design, a pedagogical approach that intentionally sequences learning activities so that students are supported to move from foundational knowledge toward more complex understanding and independent application. In this review, scaffolded designs typically progressed through stages such as preparatory knowledge-building (e.g., lectures or readings), applied engagement (e.g., role plays, community-based projects), and opportunities for reflection or extension (e.g., debriefings, assessment tasks, or advocacy planning).

Peer learning emerged as a consistent instructional feature across all included studies. While the structure of peer interaction varied, from informal discussion groups to structured interprofessional team tasks, these experiences were interpreted to support learners in navigating professional perspectives and collectively exploring advocacy in practice. This relational dimension of learning was particularly prominent in initiatives that included collaborative project work or cross-disciplinary reflection, reinforcing the importance of co-construction in the development of advocacy capabilities.

**Table 2:** Design features of included advocacy education initiatives

Study	Pedagogical activities (as reported)	Interpreted instructional design approach*	Community involvement*	Duration	Advocacy orientation*
Adams et al. (2023)	· Lectures · Workshop · Panel discussion ·Team-based learning activity · Networking session	· Didactic · Peer learning · Team-based inquiry	Unclear	1 day	Mixed
Eaton et al. (2017)	· Asynchronous readings and videos · Group discussion · Advocacy activity with other students · Student presentations	·Asynchronous E-Learning · Peer learning · Team-based inquiry	Unclear	4 h (2014), 5 h (2015) (excluding asynchronous activities)	Activism
Hallowell et al. (2024)	· Asynchronous modules · Seminars ·Advocacy project with other students and community partner · Student presentations	·Asynchronous E-Learning · Didactic · Project-based · Peer learning	Yes	8 weeks	Activism
Langlois et al. (2016)	·Semi-structured interviews with ‘health mentors’ (individuals living with chronic health conditions) · Written reflection · Online discussion	· Peer learning · Reflective practice ·Asynchronous E-Learning	Yes	6 h (excluding asynchronous activities)	Agency
Liller et al. (2020)	· Asynchronous modules · Workshop ·Advocacy activity with other students · Student presentations	·Asynchronous E-Learning · Didactic · Team based inquiry · Peer learning	No	5.25 h	Activism
Machin et al. (2014)	· Lectures/seminars · Workshop · Group project · Student presentations · Written reflection	· Didactic · Project-based · Peer learning · Reflective practice	Yes	Unclear	Mixed
Peluso et al. (2013)	· Lectures · Debate · Panel discussion · Advocacy project · Student presentations	· Didactic · Peer learning · Project-based	Yes	4 weeks	Activism
Pinto et al. (2018)	· Lectures/workshops · Community site visits ·Advocacy project with other students · Student presentations	· Didactic ·Observership/field trip · Project-based · Peer learning	Yes	1 year	Activism
Yamaoka et al. (2019)	· Seminars · Project · Student presentations · Community site visits ·Community placement/practicum	· Didactic · Project-based · Peer learning · Apprenticeship ·Observership/field trip	Yes	9 months	Mixed

* Columns marked with an asterisk reflect reviewers’ interpretations and were not explicitly stated in the included studies

#### Community involvement

A prominent feature across several studies (Hallowell et al., 2024; Peluso et al., 2013; Yamaoka et al., 2019; Langlois & Lymer, 2016; Pinto et al., 2018; Machin & Jones, 2014), was the involvement of community members and organisations in the design and delivery of advocacy education. This involvement ranged from guest lectures, workshops, or panel discussions (Peluso et al., 2013; Yamaoka et al., 2019; Pinto et al., 2018); being interviewed by students (Langlois & Lymer, 2016); hosting students during community site visits (Yamaoka et al., 2019; Pinto et al., 2018); assessing student performance (Machin & Jones, 2014); and partnering with students on advocacy projects (Hallowell et al., 2024). Six studies included clearly described community engagement, while in others the extent of involvement was limited or unclear (Table 2). This variability speaks not only to differing program aims and resourcing, but also to how community partnerships are conceptualised within curricula, either as one-off contributions or as sustained, reciprocal relationships.

#### Advocacy type and focus

While all included initiatives aimed to teach advocacy, they varied significantly in how advocacy was defined, contextualised, and prioritised. There was notable variability in the specific content and elements of the education initiatives in the included studies. In some studies, advocacy teaching was contextualised, such as advocacy specific to disability (Adams et al., 2023), however, in most studies, it was presented with a broad or non-contextual focus, emphasising general principles of advocacy application across diverse settings (Eaton et al., 2017; Hallowell et al., 2024; Liller et al., 2020; Peluso et al., 2013; Langlois & Lymer, 2016; Pinto et al., 2018; Machin & Jones, 2014).

Drawing on Hubinette et al. typology, advocacy was interpreted in the studies to reflect three broad orientations: activism (e.g., addressing systemic injustice) (Eaton et al., 2017, Hallowell et al., 2024, Liller et al., 2020, Peluso et al., 2013, Pinto et al., 2018), agency (e.g., acting of behalf of individuals) (Langlois & Lymer, 2016), or a blended approach that included both (Adams et al., 2023; Yamaoka et al., 2019; Machin & Jones, 2014) (Table 2). These orientations were inferred from the content, learning objectives, and student activities described in the studies. However, few studies explicitly defined the type of advocacy being taught, and the distinction between orientations was often implicit, with overlapping elements present across initiatives.

Themes 2 and 3 build upon the curriculum design analysis by synthesising the outcomes of the advocacy curricula, exploring how students experienced and responded to the advocacy education initiatives. Across the included studies, outcome reporting varied in type and depth. One study reported only quantitative outcomes (Eaton et al., 2017), two studies reported solely qualitative outcomes (Langlois & Lymer, 2016; Machin & Jones, 2014), and six studies employed a combination of both (Adams et al., 2023; Hallowell et al., 2024; Liller et al., 2020; Peluso et al., 2013; Yamaoka et al., 2019; Pinto et al., 2018) (Table 2). Data collection methods included post-initiative surveys (*n* = 6), pre- and post-initiative surveys (*n* = 1), student reflections and written artefacts (*n* = 3), focus groups (*n* = 1), and interviews with community partner organisations (*n* = 1) (Appendix 2). Most surveys were self-developed, with all but one study (Peluso et al., 2013) providing a clear description of outcome measures. Only one study, Eaton et al. (2017) drew on a a standardised tool, the Political Astuteness Inventory, and incorporated pre-initiative data collection. While such approaches can support comparative analysis, their presence within an otherwise qualitatively oriented body of literature highlights the diversity of evaluative strategies rather than serving as a benchmark of rigour.

### Theme 2: Impact of advocacy curriculum on student engagement and growth

This theme focuses on how advocacy initiatives shaped students’ engagement, learning, development, and future orientation. It brings together students’ responses to the initiatives and the personal and professional learning outcomes they reported. This theme is further detailed in the following subheadings.

#### Student satisfaction and perceived relevance

This subtheme captures how students experienced the initiative in terms of enjoyment, satisfaction, and perceived alignment with their academic or professional goals. It includes high satisfaction scores, positive feedback about the initiative’s structure, and the perceptions of high relevance to students’ future roles.

Students consistently reported high levels of satisfaction with the advocacy initiatives. Quantitative data from several studies reported mean satisfaction scores frequently exceeding 4.5 on a 5-point scale (Pinto et al., 2018) or 9 on a 10-point scale (Yamaoka et al., 2019). Similarly, strong endorsement of the initiatives was reflected by Liller et al. (2020), who found that 100% of participants would recommend their initiative to other students.

Qualitative responses also underscored the initiatives’ alignment with students’ professional identity. Across three studies, students viewed the content as applicable (Adams et al., 2023), important (Hallowell et al., 2024), and valuable (Peluso et al., 2013) to their anticipated practice. Alignment between advocacy education and professional aspirations was also reflectively quantitatively across included studies, with Peluso et al. (2013) reporting that 80% of participants indicated that they planned for advocacy to be an important part of their future work, and Yamaoka et al. (2019) reporting that 84.7% of participants agreed or strongly agreed that the initiative was relevant to their long-term career.

#### Development of advocacy knowledge and skills

In addition to favourable perceptions of the learning experience, students described meaningful growth in their advocacy-related knowledge and skills. Four studies reported quantitative outcomes regarding knowledge and skill in advocacy (Adams et al., 2023; Eaton et al., 2017; Liller et al., 2020; Yamaoka et al., 2019). Adams et al. (2023) and Liller et al., (2020) reported similar outcome measures, finding high levels of student agreement in a self-report survey item regarding increased knowledge and skill pertaining to advocacy following the initiative. By comparison, Eaton et al. (2017) utilised an established measurement tool as a proxy for advocacy knowledge and skill, the political astuteness inventory (PAI) as a proxy tool to quantify student knowledge and skill, and found a statistically significant increase in mean scores following the education initiative. Yamaoka et al. (2019) reported that participants’ knowledge regarding initiative content increased more than 2 points on average (on a 10 point scale). However, as advocacy was one of multiple components in this initiative, and Yamaoka et al. (2019) did not disaggregate this outcome amongst the different components of the initiative content, it is unclear if advocacy-specific knowledge increased. All studies which reported qualitative outcomes regarding ‘knowledge and skill in advocacy’ found student knowledge and skill in advocacy increased as a result of the initiative (Langlois & Lymer, 2016; Pinto et al., 2018; Machin & Jones, 2014). 

#### Advocacy identity and future intentions

Several studies revealed that participation in the initiatives also contributed to a developing advocacy identity (Peluso et al., 2013; Yamaoka et al., 2019), expressed through student intention to incorporate advocacy into their future roles, as well as active engagement with advocacy subsequent to advocacy curricula. Peluso et al. (2013) reported that 80% of students subsequently intended for advocacy to be a significant component of their future work, and that some students engaged with advocacy material beyond the classroom (Peluso et al., 2013), with the authors suggesting that “the experience had fostered an awareness of and enthusiasm for this aspect of physician practice” (p.72). In addition, a small number of students continued their advocacy projects after the formal teaching period or became involved in institutional advocacy groups (Peluso et al., 2013). Similarly, Yamaoka et al. (2019) reported that 43% of respondents were involved in advocacy following the advocacy education initiative. While such behavioural outcomes were less commonly documented, they offer an important signal that advocacy education can inspire longer-term commitment and potentially shape professional identity formation.

### Theme 3: Student perspectives on advocacy curriculum: enablers, challenges

This theme groups together the relational, organisational, and pedagogical elements that influenced how students experienced the initiatives. It recognises both what supported engagement and what introduced tension or complexity in the learning process.

#### Teaching methods and instructional design

Across the reviewed studies, students expressed strong appreciation for interactive, varied, and learner-centred teaching methods. Three studies provided quantitative outcomes regarding perceived effectiveness of the initiative or its components (Adams et al., 2023; Hallowell et al., 2024; Liller et al., 2020), all reporting overall participant agreement that the initiative and/or its elements were effective in their implementation. Hallowell et al. (2024) reported average teaching effectiveness scores of 4.5 or greater (on a 5 point scale) across the survey items, with the exception of one item regarding orientation to the education initiative (mean = 3.9) (Hallowell et al., 2024). Hallowell et al. (2024) also highlighted that a survey item regarding the value of working with community organisations received a particularly high score of 4.8. Comparing perceived effectiveness across teaching methods, Adams et al. (2023) reported higher effectiveness for panels, lecture-based sessions, team-based learning sessions and workshops (all greater than 90%), compared to the networking session (64.7%).

Mixed modalities, such as case-based learning, seminars, team-based activities, and experiential projects, were described as engaging and effective. Pinto et al. (2018) reported that students appreciated mixed theoretical and practical teaching, however expressed a desire for “greater emphasis on case studies and more clinically-oriented experiental learning” (p.128). Students particularly appreciated interactivity within the sessions (Adams et al., 2023), and high levels of discussion and participation within the interprofessional setting (Peluso et al., 2013).

There were also implementation considerations which emerged that were unique to certain teaching methods. For example, Peluso et al. (2013) reported that the structured debate activity received poorer feedback compared to other teaching methods in the initiative, and speculated this was “likely due to the fact that students were assigned to positions in the debate rather than encouraged to express their own personal opinions.” (p.72) The use of project-based learning emerged as a double-edged sword. Three studies reported outcomes related to project-based instructional approaches, and found that students encountered challenges with completing their projects (Machin & Jones, 2014), and expressed the need for greater clarity (Hallowell et al., 2024) and support (Pinto et al., 2018). Therefore, while project-based learning promoted critical thinking and real-world engagement, students in several studies reported challenges related to unclear expectations, insufficient scaffolding, and a lack of support to complete projects.

#### Interprofessional learning dynamics

This subtheme explores how students experienced learning with peers from other health professions. It includes positive outcomes such as enhanced appreciation of diverse perspectives, but also instances of tension or conflict arising from professional role boundaries. Many students highlighted the benefits of working with peers from other health disciplines (Hallowell et al., 2024), noting that the interprofessional nature of the initiatives enriched their understanding and skill regarding advocacy through exposure to diverse perspectives and collaborative dialogue (Machin & Jones, 2014). Indeed, participants described both greater role awareness, learning about “different professional roles in the health service, the work they’re responsible for, difficulties they face, policy drivers and systems they work in” (Machin & Jones, 2014, p.221), as well as practical skills in collaborative advocacy practice, where, for example, “overcoming traditional professional differences can be achieved through a joint focus on service user need” (Machin & Jones, 2014, p.221). One study reported that interprofessional collaboration also introduced unexpected tension, with students experiencing conflict regarding “justifications behind individual and population approaches to health” (Peluso et al., 2013, p.72) and disagreements regarding the “contributions of clinical and public health practitioners to the problems and solutions discussed” (Peluso et al., 2013, p.72).

#### Community partnerships and real-world engagement

Engagement with community organisations and individuals with lived experience was another frequently cited strength. Students described these interactions as “the best aspects of the course” (Pinto et al., 2018, p.128), with another study highlighting that students reported community collaboration was crucial “for their understanding of how to work with communities” (Hallowell et al., 2024, p.205), and thereby enabling practical experience of advocacy practice as a partnership process As discussed in Theme 1, in some cases, community partners not only contributed to teaching but also evaluated student work or collaborated on advocacy projects. One study noted that student efforts were of practical benefit to the community organisations involved (Hallowell et al., 2024).

#### Barriers to learning and implementation

Despite generally positive responses, students and educators also encountered barriers that impacted the effectiveness of the initiatives. Beyond the challenges of project-based learning, other limitations included inadequate guidance, unclear task requirements, and uneven quality of facilitation. In some contexts, interprofessional tensions and instructional mismatches between disciplines were noted as contributing to learning challenges. Debate formats were perceived to constrain students’ ability to express nuanced or personally held perspectives, limiting the depth of engagement, whereby “participants did not see this format as a success”, despite the researchers’ stated intention “to have students study and understand positions that they might one day advocate against” (Peluso et al., 2013, p.72).

## Discussion

This narrative review synthesised nine studies describing interprofessional education (IPE) initiatives in health professions education (HPE) that were designed to deliver focused advocacy curricula. The findings are organised around three interconnected themes: curriculum design, impact of advocacy curriculum on student engagement and growth, and student perspectives on advocacy curriculum: enablers, challenges. Together, these themes offer insight into how advocacy education was conceptualised, delivered, and experienced in IPE settings, while also identifying consequences for implementation and areas for future development.

The included studies demonstrated wide variation in the content, structure, and duration of advocacy initiatives. Teaching methods ranged from lectures and workshops to project-based learning, community site visits, and asynchronous e-learning. When synthesised into overarching instructional approaches, a consistent pattern of scaffolded learning emerged. These scaffolded models progressed through stages of knowledge acquisition, application, and extension, aligning with broader educational literature supporting staged learning design in IPE, where increasing complexity as students’ progress may facilitate the transfer of learning to new and different situations or contexts (Hean et al., 2012). This aligns with broader claims that interprofessional education can mirror the team-based nature of advocacy in real-world settings.

Community partnerships were a recurring and valued feature, with organisations and individuals with lived experience participating in varied capacities, from delivering lectures and hosting visits to co-developing or assessing student work. These interactions grounded learning in lived experience and social context, offering a situated perspective to students’ learning and were often described as the most meaningful components of the initiatives. This finding aligns with existing literature that supports the importance of experiential learning in advocacy curriculum in HPE (Morris et al., 2019; Daya et al., 2021). The review also supports assertions in the literature that collaborative approaches to advocacy education, particularly those underpinned by sustained community partnerships, may not only offer students practical experience of advocacy as a relational activity undertaken *with* others rather than *for* others (To & Sharma, 2015; Hubinette et al., 2015) but also foster reciprocal learning, mutual benefit, and material gains for community partners (Truong et al., 2023). However, the processes underpinning community collaboration were inconsistently reported, making it difficult to evaluate the equity, sustainability, or mutuality of these partnerships. This under-reporting highlights the need for future studies to more fully describe how community engagement is initiated, maintained, and evaluated.

Students’ feedback pointed to clear benefits of the IPE format in learning advocacy (Hallowell et al., 2024; Peluso et al., 2013; Machin & Jones, 2014). Collaboration across disciplines enriched the educational experience by exposing students to diverse perspectives and promoting an understanding of advocacy as an inherently collective and negotiated process. This finding therefore supports propositions within the literature that IPE may provide a unique opportunity and effective method for students to learn about advocacy in a way which reflects the interprofessional and collaborative approach to advocacy seen in practice (To & Sharma, 2015). However, interprofessional dynamics also presented challenges. In one study, conflict emerged due to differing assumptions between disciplines (Peluso et al., 2013), yet this tension may reflect real-world complexities of collaborative advocacy, offering valuable learning if appropriately scaffolded. This duality underscores the importance of skilled facilitation and attention to group process within IPE advocacy contexts.

Students also described increases in advocacy knowledge and skills, particularly in navigating systems, engaging with stakeholders, and articulating change efforts. One study using the Political Astuteness Inventory demonstrated measurable gains in advocacy capability, while others reported strong self-assessed development. Importantly, a small number of studies tracked students’ intentions and actions beyond the formal learning experience. Some students continued advocacy projects or joined related groups, suggesting that well-designed education may contribute to the development of a professional identity that incorporates advocacy knowledge, skills, and commitments. These findings resonate with recent calls to evaluate advocacy education not just through knowledge metrics, but also through behavioural and identity-based outcomes (Howell et al., 2019; Agrawal et al., 2023).

Despite these successes, the findings from this narrative review also draw attention to the broader challenges of IPE advocacy education. Students encountered barriers that highlight the need for careful educational design. In particular, project-based and experiential learning methods, while highly valued, were sometimes undermined by unclear expectations or inadequate support. Without thoughtful scaffolding, these otherwise potentially transformative approaches risk leaving students confused or disengaged. Similarly, when students were asked to adopt advocacy positions in structured debates that did not reflect their values, this created discomfort and detracted from the learning experience. Poorly executed, inadequate or inappropriate assessment may risk trivialising health advocacy knowledge or skills, privileging that which is easy to assess over that which is meaningful (LaDonna et al., 2023). These examples reinforce that effective advocacy education requires both pedagogical ambition and practical support.

The interprofessional context of the included initiatives also shaped the type of advocacy addressed. Most studies focused on activism or a mix of activism and agency, with fewer addressing agency alone. This contrasts with trends in discipline-specific literature (McDonald et al., 2019), where agency is more commonly prioritised. The emphasis on collective forms of advocacy within IPE may reflect the suitability of interprofessional settings for exploring systemic change. However, this also raises questions about how different types of advocacy are best taught, assessed, and supported within varied educational environments.

Findings from this narrative review indicate the potential for advocacy education in IPE contexts to be rich, transformative, and professionally generative, particularly when supported by deliberate design, authentic engagement, and structural alignment. These findings suggest that advocacy should not be confined to isolated curricular moments (Hubinette et al., 2017) but woven throughout the educational program in ways that are intentionally aligned with institutional commitments to social accountability, including mission, values, and community engagement.

### Strengths and limitations

Comprehensive search and data collection strategies were used, and the resulting synthesis provides an overview of current areas where advocacy and IPE intersect in the context of HPE. However there are some limitations with the search strategy, as it is possible that publications which did not explicitly include ‘advocacy’ or ‘advocate*’, but still explored related topics consistent with our definition of advocacy, were missed and not assessed for eligibility. However, the high yield returned from the search (*n* = 941) and large number of full-texts reviewed (*n* = 131) does increase confidence in the search strategy. Finally, generalisability of this review is limited by the small number of countries represented across the included studies, as well as exclusion of studies conducted in the workplace rather than university setting.

There were also some limitations specific to the included studies themselves. For included studies where advocacy formed one of multiple other primary components of the initiative (Liller et al., 2020; Yamaoka et al., 2019; Langlois & Lymer, 2016; Pinto et al., 2018; Machin & Jones, 2014), findings which were applicable to the initiative as a whole may not be representative of the advocacy component specifically. The heterogeneity of outcome reporting across the included studies limited the ability to compare or draw conclusions regarding the efficacy of the various IPE initiatives described.

### Implications for education

This review highlights implications for educational practice and curriculum development. Firstly, IPE appears to be well-placed for teaching advocacy to health professions students in a way which reflects the interdisciplinary practice of advocacy, and facilitates understanding and experiencing advocacy as inherently collaborative. Secondly, embodying the collaborative principles of advocacy in education may benefit significantly from community partnership. Thirdly, scaffolding diverse and interactive instructional approaches may be useful in constructing an integrated and holistic interprofessional advocacy curriculum.

### Implications for further research

Evaluation of interprofessional advocacy education initiatives often focus on self-reported gains in knowledge and skills; however, broader and more diverse outcome measures may strengthen future evaluation. There is a clear need to develop consistent conceptualisations of advocacy and to address the challenges inherent in assessing advocacy, particularly in interprofessional settings. Future curriculum design would benefit from striving for longitudinal outcomes, co-design processes with community partners, and including ways in which interprofessional identity and collaborative competence intersect with advocacy development. As advocacy becomes an increasingly recognised competency in HPE (Bessette et al., 2020; Agrawal et al., 2023; Blavos et al., 2024; Gandra & Silva, 2023), the lessons drawn from these initiatives can inform more inclusive, impactful, and sustainable curriculum design.

There is a need for consistent and contemporary conceptual frameworks for health advocacy, particularly those that reflect the complexity of advocacy within interprofessional and global contexts. Such frameworks would help to guide the design, delivery, and assessment of advocacy curricula in health professions education. Future studies should also explore the challenges of assessing advocacy competence, including the need for credible assessment criteria and recognition of advocacy work that often occurs outside formal learning environments. By addressing these gaps, future research can strengthen the foundations for meaningful, sustainable advocacy education.

## Conclusion

This narrative review synthesised nine interprofessional education (IPE) initiatives designed to deliver advocacy education within health professions programs. Despite substantial variation in scope, structure, and delivery, common elements included scaffolded, experiential learning, and community engagement. Student responses highlighted perceived relevance, and the value of interprofessional and community-based learning. However, challenges such as instructional mismatches and interprofessional tensions were also noted. Across the studies, advocacy education was most impactful when it facilitated relational learning, critical reflection, and connections to lived experience. Moving forward, the field would benefit from conceptually grounded and contextually adaptable definitions of advocacy that reflect interprofessional practice. Further research is needed to develop diverse evaluation strategies, beyond satisfaction and self report, that capture students’ evolving identities, actions and sustained advocacy engagement. Ultimately, strengthening conceptual clarity and assessment practices will be critical for designing curricula that prepare graduates to engage meaningfully in advocacy throughout their professional lives.

## Supplementary Information

Below is the link to the electronic supplementary material.

Supplementary Material 1

Supplementary Material 2

## Data Availability

No datasets were generated or analysed during the current study.
