# Supplementary material for: Interprofessional education initiatives to teach advocacy in health professions programs: a narrative review
Source: Adv Health Sci Educ Theory Pract. 2026 Jan 22;31(4):1399–418. doi: 10.1007/s10459-026-10502-6 (PMC13553672; doi:10.1007/s10459-026-10502-6)
Supplement: Supplementary file 1 [file 10459_2026_10502_MOESM1_ESM.docx]

# Appendix 1 - Search strategies

|  | **DATABASE 1:**  **OVID (MEDLINE + EMBASE + PsychInfo)** | **DATABASE 2:**  **ERIC** | **DATABASE 3:**  **CINAHL** |
| --- | --- | --- | --- |
| **Interprofessional education**  **AND** | interprofessional education OR  interprofessional teaching OR  interprofessional learning OR  interprofessional training OR  interdisciplinary education OR  interdisciplinary teaching OR  interdisciplinary learning OR  interdisciplinary training OR  multidisciplinary education OR  multidisciplinary teaching OR  multidisciplinary learning OR  multidisciplinary training OR  inter-professional education OR  inter-professional teaching OR  inter-professional learning OR  inter-professional training OR  inter-disciplinary education OR  inter-disciplinary teaching OR  inter-disciplinary learning OR  inter-disciplinary training OR  multi-disciplinary education OR  multi-disciplinary teaching OR  multi-disciplinary learning OR  multi-disciplinary training | interprofessional education OR  interprofessional teaching OR  interprofessional learning OR  interprofessional training OR  interdisciplinary education OR  interdisciplinary teaching OR  interdisciplinary learning OR  interdisciplinary training OR  multidisciplinary education OR  multidisciplinary teaching OR  multidisciplinary learning OR  multidisciplinary training OR  inter-professional education OR  inter-professional teaching OR  inter-professional learning OR  inter-professional training OR  inter-disciplinary education OR  inter-disciplinary teaching OR  inter-disciplinary learning OR  inter-disciplinary training OR  multi-disciplinary education OR  multi-disciplinary teaching OR  multi-disciplinary learning OR  multi-disciplinary training | interprofessional education OR  interprofessional teaching OR  interprofessional learning OR  interprofessional training OR  interdisciplinary education OR  interdisciplinary teaching OR  interdisciplinary learning OR  interdisciplinary training OR  multidisciplinary education OR  multidisciplinary teaching OR  multidisciplinary learning OR  multidisciplinary training OR  inter-professional education OR  inter-professional teaching OR  inter-professional learning OR  inter-professional training OR  inter-disciplinary education OR  inter-disciplinary teaching OR  inter-disciplinary learning OR  inter-disciplinary training OR  multi-disciplinary education OR  multi-disciplinary teaching OR  multi-disciplinary learning OR  multi-disciplinary training |
| **Advocacy** | advocacy OR  advocate* | advocacy OR  advocate* | advocacy OR  advocate* |
