# Supplementary material for: Interprofessional education initiatives to teach advocacy in health professions programs: a narrative review
Source: Adv Health Sci Educ Theory Pract. 2026 Jan 22;31(4):1399–418. doi: 10.1007/s10459-026-10502-6 (PMC13553672; doi:10.1007/s10459-026-10502-6)
Supplement: Supplementary file 2 [file 10459_2026_10502_MOESM2_ESM.docx]

# Appendix 2 - Summary of interprofessional education initiatives to teach advocacy in health professions programs across included studies

| **Authors, year and country** | **Study design** | **Participants** | **Intervention (education initiative)** | | | **Outcome** | | | **Author's conclusion** |
| --- | --- | --- | --- | --- | --- | --- | --- | --- | --- |
|  |  |  | **Teaching method** | **Frequency and duration** | **Elements** | **Data collection method** | **Outcomes measured** | **Results** |  |
| Advocacy only component of initiative* | | | | | | | | | |
| Elizabeth J. Adams, Samantha Schroth & Trisha Kaundinya; 2023; United States | Post intervention study without controls | n=30 total participants (24 respondents)  7 health disciplines: audiology, genetic counselling, medicine, medical scientist, nursing, prosthetics and orthotics, public health.    Note: total participants also included ‘other’ disciplines. | Interactive virtual conference including lectures, workshops, panels, networking session, and a team-based learning activity. | 1 day | "The 2021 conference theme was ‘Advancing Disability Equity in Medicine’"    "The goal of the DAC Med conference was to raise awareness of the disparities and challenges those with disabilities face in healthcare from a patient and  provider perspective, as well as to provide a primer on the training needed to deliver dignified care to patients living with a wide range of disabilities."    "Session titles included:  - Disability Inclusion in Research and Health Curricula  - Multidisciplinary Spheres of Disability Advocacy: A Panel  - Networking Lunch Hour Breakout Rooms  - Healthcare Disparities for Patients with Disabilities and Provider Bias  - Introduction to American Sign Language for Healthcare Workshop  - Introduction to Accessible Equipment Workshop  - Team-Based Disability Management in Healthcare: Case Based Learning  - Disability Visibility in Narrative: A Trainee Panel" | Post-conference student survey: “combination of 5-point Likert scale-based questions and free response questions.” | “Self-reported demographics, experience in disability advocacy, curricular exposure to disability education, impact of the virtual conference, and perceived effectiveness of each conference session.” | “Background in disability advocacy  The majority of participants (58.3%) did not believe they had a strong background in disability rights or advocacy before attending the conference, with 26.1% learning about ableism in healthcare through their program’s curriculum. Half (50.0%) of participants attended health professional programs that introduced the Americans with Disability Act (ADA) in their curricula. Intersectionality of disability with other minoritized identities was similarly covered in 48% of respondents’ curricula. A total of 12.5% of survey respondents agreed that they had sufficient resources to provide care to patients with disabilities before attending the conference.”    “Impact of single-day conference learning  Almost all attendees (91.6%) reported participating in the DAC Med conference to learn how to be a better advocate and ally for patients and peers with disabilities in healthcare, with 95.8% reporting that the conference provided them with this knowledge. A total of 87.5% of participants agreed that they acquired additional resources to better care for patients with disabilities. Ninety-two percent of respondents reported that attending the DAC Med conference furthered their background in disability rights and advocacy. Most attendees agreed the different conference sessions were effective, including the panels (96.9%), lecture-based sessions(95.3%), team-based learning session (94.7%), work-shops (92.5%), and networking session (64.7%). In a free response survey question, respondents noted that they appreciated how interactive the conference sessions were and the applicability of covered topics” | "Single-day virtual, interactive conferences are effective in beginning to address these gaps by providing additional resources and empowering students to employ them. Future interprofessional, student-driven educational opportunities should be implemented to provide leaners with additional training level-appropriate opportunities to engage in educational and advocacy-based work in the disability sphere." |
| Melody Eaton, Maria deValpine, Julie Sanford, Jamie Lee, Kandy Smith; 2017; United States | Pre and post intervention study without controls | n=82 (2014)  n=265 (2015)  4 health disciplines: nursing, health sciences/administration, social work, communication sciences and disorders | - Readings and short videos including core knowledge needed for the activity.  - Case study exercise completed individually, then in small groups, followed by a group discussion.  - Small group activity: ‘mind the gap’ policy exercise, which included producing posters.  - Posters were displayed and voted on by peers and state legislators.  - The top 3 posters were presented by the winning participant groups, followed by a discussion with the legislators. | 4 hours (2014)  5 hours (2015) | “This study explored the effectiveness of a team-based policy advocacy exercise to improve political astuteness in undergraduate health professions students.”  “Discussion of the case study responses included lobbying and legislative insights, providing the participants with essential background information necessary for synthesis in the policy advocacy exercise that followed.”  The policy exercise involved working in small groups to develop an alternative plan to finance the gap in health insurance coverage for individuals who do not qualify for Medicaid or the Health Insurance Marketplace. | Pre and post education initiative survey | Political Astuteness Inventory (PAI) assessment tool: "40-item inventory that assesses political understanding, participation, and general knowledge of the legislative process" | "Both the 2014 and 2015 exercises demonstrated that political astuteness was significantly different after participants experienced the HPS with an interprofessional TBL policy advocacy activity, as measured using the PAI. Resulting means indicated scores increased significantly, from 9.09 (SD, 5.69) and 8.14 (SD, 4.9) before each activity to 12.0 (SD, 6.40) and 9.85 (SD, 5.85) after the summit exercises (2014 pilot: t = 2.78, P G .01; 2015 study: t = j3.59, P G .01). A Mann-Whitney test validated significance for the 2015 study regarding a smaller mean difference. Results indicated that the median political astuteness was greater post TBL activity, (U = 37.492 with n1 = 238 or the standardized test statistic of 3.838, P G .01). Moreover, both study results demonstrated a shift in political astuteness from level 1, or completely politically unaware, to level 2, slightly politically aware, after participating in the interprofessional TBL health policy advocacy activity."  "In both the pilot and the 2015 studies, there was an increase in awareness but not an increase in intention to participate in advocacy activities." | "The HPS incorporated interprofessional student TBL health policy advocacy activities using a team-based learning model. This was effective in improving student knowledge and engagement in health policy advocacy and increasing political astuteness. Improving collaborative interprofessional health policy advocacy may prove to enhance health care professionals’ likelihood of understanding and involvement. This has the potential to influence health policy in the future." |
| Ronan Hallowell, Sonali Saluja, LaVonna Lewis, Daniel A. Novak, Wenonah  Valentine, Eric Batch, Mark-Anthony Clayton Johnson, Ricky N. Bluthenthal,  Michael R. Cousineau & Ron Ben-Ari; 2022; United States | Post intervention study without controls | n=11  2 health disciplines: medicine, public health | - 3 60-90 minute asynchronous online modules  - 5 90 minute synchronous zoom sessions.  - Students worked in interprofessional groups with a community organization partner on a community project outside of the above class times.  - During the final asynchronous zoom session, the student groups presented their projects to faculty and school administrators and their community organization partners. | 8 weeks | Aims of study are to to address the following four advocacy-related competencies:  "1. How to listen to and work in collaboration with community members, stakeholders, and allies across disciplines and demographic categories;  2. How to identify advocacy opportunities from the structural and systems barriers clinicians observe in their daily work (i.e., ‘patient to policy’ advocacy);  3. How to develop a targeted advocacy strategy that has a reasonable chance for success;  4. How to utilize multiple advocacy tactics."    Asynchronous online modules included:  "(1) Principles of Health Justice and Structural Competency;  (2) Becoming an Advocate; and  (3) Critically Appraising Community Health Strengths and Needs.  These modules covered basic information about advocacy skills and the need for social, economic, and political change to ameliorate health inequities based in structural racism and the oppression of other marginalized groups."    Synchronous activities included the following:  "(1) Disrupting Injustice: Course Introduction;  (2) Integrating a Health Justice and Advocacy Framework;  (3) Setting an Agenda with Cultural Humility;  (4) Translating Community Needs to Advocacy Strategies; and  (5) Final Project Presentations"    Community organisation partner projects included:  "(1) Black maternal mortality;  (2) alternatives to law enforcement in non-custodial hospital settings; and  (3) a California legislative initiative to repeal a ban on the ability of municipalities to impose soda taxes" | “9-item survey using a 5-point rating scale and one-short answer” at end of course    Qualitative thematic analysis of: short answer survey question, student focus groups, individual interviews with community organisation partners, student reflections as part of asynchronous online modules | Survey: N/A    Qualitative thematic analaysis: N/A | Survey results:  "Students were, overall, very satisfied with the course with eight items that asked about details of the course implementation having a mean score of 4.5 or higher. They found the opportunity to work with community organizations very valuable and on average rated the statement, “Working with the community organization was a valuable learning experience,” a 4.8 (SD = 0.40). One item that asked if, “The orientation to course activities was sufficient to provide the information needed to complete the coursework and project,” had a mean score of 3.9 (SD = 1.14)."    Qualitative thematic analysis findings:  - Definition of projects  "Students expressed a desire for a better sense of what they were being asked to deliver to the community organization at the beginning of the course."  -Student group and liaison communication  "The three groups varied in their ease of communication within their student groups and with their community liaison. The students understood that the community liaisons’ schedules sometimes resulted in delayed communication. One student suggested that an alternate/back-up liaison from each organization could have been helpful for when the primary liaison was unavailable. The communication within the groups varied."  - Interdisciplinary collaboration  "The qualitative feedback indicated that both MD and MPP students valued collaborating with and learning from their counterparts in the course."  - Community collaboration  "MD student [redacted] acknowledged that what they learned was important for their understanding of how to work with communities."  "MD student [redacted] noted how allying and collaborating with patients as well as other professionals in lobbying for change is crucial and that the course gave them a new view on the possibility of changing policy."  "The community partners indicated that the students consultative work products were useful for their initiatives, and that they found working with MD and MPP students to be a valuable way to think about how to build stronger and more inclusive coalitions to advocate for health justice." | "The interdisciplinary and community collaboration elements of the project were educative, useful to the community organizations, and provided meaningful opportunities for students and community members to get to know each other in productive ways. The template we provide in this article and the curriculum blueprint...can  be leveraged by other schools to expand their efforts in preparing students to serve their communities with cultural humility, amplify the strengths of the communities they seek to serve, and respond to the needs that communities themselves have prioritized. This learning approach and structure of the Advocacy for Health Justice course can serve as an enduring and scalable method to promote the development of future physicians and those trained in public policy to be better able to identify and implement advocacy interventions that could mitigate health inequities affecting patients, communities, populations, and the health system as a whole." |
| Advocacy one of multiple components of initiative* | | | | | | | | | |
| Michael Peluso, Brian Seavey, Gregg Gonsalves, and Gerald Friedland; 2013; United States | Post intervention study without controls | n=30 total participants (19 respondents)    4 health disciplines: medicine, nursing, public health, and the physician associate programs | 4 synchronous sessions consisting of a lecture, a debate, an activist panel discussion, and presentations of students' advocacy projects.    Advocacy project regarding an issue of interest. | 4 weeks | Overall theme: ‘Advocacy and Activism in Global Health’    "The objectives of the module were to provide students with the knowledge to discuss the successes and failures of health activism in the United States and abroad and the skills to develop their own advocacy projects on an important issue facing underserved populations. A key guiding principle of the module was its foundation in inter-professional education.”    “The module had four sessions: an introduction to the history of health activism; a faculty-moderated debate on ‘current controversies in health and development’ focused on funding for AIDS programs; a panel that allowed students and activists to discuss the successes and failures of health advocacy; and student presentations of their advocacy projects. These projects required students to identify an issue of interest, engage in advocacy using skills developed during the module, and present their topic and progress to a group of students and faculty." | End of module survey | Unclear | "80% of participants stated that they planned for advocacy to be an important component of their future work, suggesting that the experience had fostered an awareness of and enthusiasm for this aspect of physician practice.  Students completed advocacy projects in the following areas: hepatitis B, maternal health in the Peruvian highlands, a local disease prevention day, sexual violence and post-exposure prophylaxis access, tuberculosis and the mining industry in South Africa, and access to essential medicines.  Some participants continued to pursue their projects after completion of the module. For example, one student developed his project into a ten-week research experience and has since directed a documentary film on the issue.  Several participants became involved with advocacy groups at our institution."    "Students felt that there was a high level of discussion and participation in the module setting, and that they had excellent access to the module faculty."  They were likely to engage with the material outside of class and felt that the program was a valuable supplement to the formal curriculum."    "While students rated positively the introductory, advocate panel, and student presentation sessions, the structured debate received poorer reviews. This was likely due to the fact that students were assigned to positions in the debate rather than encouraged to express their own personal opinions. The intention was to have students study and understand positions that they might one day advocate against but the participants did not see this format as a success."  An unexpected result of the inter-professional module was a surprising tension during discussions between the students of clinical medicine and public health. This included conflict regarding both the justifications behind individual and population approaches to health and disagreements regarding the contributions of clinical and public health practitioners to the problems and solutions being discussed." | "The inclusion of medicine, physician associate, public health, and nursing students broadened the discussion and emphasized the collaborative, inter-professional nature of health advocacy. While individuals from these disciplines will work together on healthcare teams throughout their careers, the module represented a unique opportunity to collaborate as students. Utilizing a faculty leader with a long history in health activism but without medical training allowed students to develop a richer perspective on the internal and external views of their roles in this field. The module used a low-cost, easy-to-implement format that may be organized by interested students and faculty at any institution. It achieved a reasonable balance in terms of time allocation, as it represented a compromise between a full course and a single event with the aim of providing participants with an extended educational experience without burdening students already participating in the medical curriculum." |
| Sylvia Langlois, Erin Lymer; 2016; Canada | Post intervention study without controls | n=91    7 health disciplines: medicine, nursing, occupational therapy, pharmacy, physical therapy, social work, speech-language pathology | 4 meetings (guided by semi-structured interview questions) between interprofessional groups of five students and a health mentor (people with chronic health conditions and multiple contact points in the healthcare system) from the community.  “Following each encounter, students submitted a written reflection and participated in facilitated asynchronous online discussions to advance learning pertaining to the interprofessional communication and collaboration competencies, as well as the topic explored in the module” | 4 meetings of 90 minute duration with health mentors | The four broad program meetings/modules included:  “(1) Chronic Health Challenge in Context;  (2) Impact of the Chronic Health Challenge;  (3) Ethical and Professionalism Issues; and  (4) Patient Safety Issues.”    This paper only describes the "Ethical and Professionalism Issues" module.    Semi-structured interview questions for the Ethical and Professionalism Issues module explored: “respect, inclusion of the individual in the decision-making process, communication, perceptions of adherence to management plans, and professional and unprofessional behaviours experienced.” | Inductive thematic analsysis of student written reflections and text from asynchronous online discussions | N/A | "Five major themes emerged from the data:  (1) Patient autonomy and expertise in care;  (2) Ethical complexity as an inevitable reality of the clinical practice setting;  (3) Patient advocacy as an essential component of day-to-day practice;  (4) Qualities of remarkable clinicians informed personal ideals for future practice;  (5) Patients’ perspectives on clinician error enabled suggestions for improving future practice."    "Theme 1: Patient autonomy and expertise in care  Students viewed the value of autonomy as foundational to respect for patients’ dignity, including concepts of independence and partnership. For example, …incidents such as these emphasize to me the need for healthcare professionals to focus on patient-centered care and to understand that the relationship is a partnership between the patient and the professional, and not one telling the other what to do and the other doing it without question.  Frequently, students referred to the patient as being the “expert” in his/her care. Students upheld the belief that the patient should be consulted and that he/she should be provided with as much information as possible to make informed decisions, which in turn validates their autonomy.  "In one incident she advised a resident physician to wait before running X-rays on her daughter following her surgery. He thought she was completely out of line; when in fact she is the expert in her daughter’s care and knows how she typically recovers following surgery. The supervising physician took this opportunity to teach the resident how important it is to listen to your patients and understand that they are the experts in their care; they know themselves best.""    "Theme 3: Patient advocacy as an essential component of day-to-day practice:  *Student B*  Students emphasized the importance of empowering patients to advocate for themselves and the importance of being advocates for individuals when their concerns are not heard or needs not met. Students described the perceived relationship between providing sufficient information to patients and enabling them to advocate for themselves. They highlighted the importance of advocating for patients whose disabilities inhibited self-advocacy. "…I will speak up and advocate for my clients and let other healthcare professionals know that this is a very important concept, and displaying compassion and respect for the person’s autonomy is crucial…"  More broadly, they discussed the importance of advocacy in the community, advocacy to service providers and program administrators, and advocating for patients to colleagues within the healthcare team.  I would try to find out what kinds of support networks my client has in their community and what gaps there may be. I would try to connect him or her with a social worker or suggest support groups that may help with coping strategies for aspects of their illness." | "This study revealed that students did develop an appreciation for patient autonomy and considered ethical dilemmas in practice settings. Of particular note is student recognition of their responsibility to advocate for patients within the healthcare team and in the broader community. With enhanced understanding of the impact of good and poor professional behaviours on individuals, students voiced commitments to ensuring that they would practice in a manner where patients would have a voice. These findings could be explored further to investigate potential integration of patient experiences into teaching of professional ethics" |
| Karen D. Liller, Zachary Pruitt and Somer Goad Burke; 2020; United States | Post intervention study without controls | n=30 total participants (13 respondents)    4 health disciplines: public health, nursing, pharmacy, and physician assistant | - 2 45 minute asynchronous modules on policy development  - 1 45 minute asynchronous module on advcoacy and coalition development  - 1 3 hour synchronous online interactive activity session, including 1.5 hour review of module content, and 60-90 minutes for breakout group activity where participants prepared advocacy brief and later presentated to other participants    Components of the advocacy brief included:  - What is the issue?  - Magnitude of the problem  - Best practices available anywhere  - Recommendations  - Impact/consequences statement  - Summary | 5.25 hours | "Objectives of the two health policy modules were:  (1) understand the relationship of federal and state/local governments in public health law,  (2) recognize the dichotomy of common good vs. individual freedoms in public health law,  (3) summarize the steps involved in the development of policy, and  (4) explain the phases of health policymaking.”  “Objectives for the advocacy and coalition building module were:  (1) define the similarities and differences between advocacy and lobbying for public health issues;  (2) explain best practices for effective advocacy including communication and meeting skills with legislators; and  (3) describe steps that will build effective coalitions for advocacy efforts.  Topics covered in the advocacy module were definitions of advocacy which can be any person or group who advocates, supports, and argues for a cause and lobbying such that a lobbyist is generally a paid representative of a group, organization, or industry who communicates with legislators about specific legislation and expresses the view or opinion of their organization; effective tips for performing successful advocacy including, knowing the difference between regular advocacy and media advocacy such that in media advocacy the target audience are those individuals who can make policy changes; and steps in building a successful coalition. These steps focus on making sure your coalition is broad-based in participation, has a mission, goals and objectives, and stays focused on the advocacy issue during meetings and in-between meetings.”  Topics for advocacy brief breakout group activity included:  “(1) universal motorcycle helmet laws, all ages, all riders;  (2) increase funding for human trafficking programs; and  (3) ban assault weapons.  These public health areas were chosen based on relevance for the geographic area and a long history of difficulties in the passage of related legislation." | Online survey with five largely Likert-type items and one text-based feedback question | Effectiveness of asynchronous modules, effectiveness of initiaitve in achieving goal, effectiveness of breakout groups in improving teamwork ability, knowledge in foundations of health policy and advocacy after initiative, whether participants would recommend program | "The respondents found the modules very effective (23%) or effective (77%) in preparation for the event. They also agreed (77% responded definitely yes, and 23% responded yes) that the modules met the goal of the session which was to use a team approach to address a current healthcare issue that could be solved with health policy changes and/or health advocacy. For the breakout sessions strengthening teamwork ability, the majority responded affirmatively, with 77% strongly agreeing and 23% somewhat agreeing to this item. Over three-quarters of the respondents (77%) strongly agreed that the event increased their knowledge of policy and advocacy, while 23% somewhat agreed. Finally, all respondents (100%) reported they would recommend the program to other students. , Students remarked that it would be beneficial if they had more time to work on their position paper. " | "Use of asynchronous modules and a synchronous session proved to be a feasible educational strategy. In concert with the tenants of adult education, this educational experience allowed adult learners to partake in curricula that was problem-centered and for students to be involved with the direction of the course through their teamwork discussion. We encourage health programs and disciplines to pursue IPEs with their students and incorporate adult education strategies." |
| Alison Machin, Diana Jones; 2014; United Kingdom | Post intervention study without controls | n=420 total participants (150 respondents)  5 health disciplines: nursing, midwifery, social work, physiotherapy and occupational therapy | - Introductory learning day regarding the theory of service improvement  - Allocation to interprofessional peer group for support  - Planning and implementation of small scale service improvement project in practice placement setting  - Assessment via oral project presentation and written reflection on learning. | Unclear | "Learning outcomes included a critical appreciation of service improvement tools such as the Plan–Do–Study–Act (PDSA) cycle.”  “Patient safety from a service user perspective was the trigger for their project”  “Students participated in an interprofessional core day focused on service improvement theory, patient safety and understanding their complementary roles in a service improvement, systems context.”  “Students subsequently identified and implemented, if appropriate and possible, a small scale, manageable improvement project in their practice placement setting. They were linked with an interprofessional buddy group for support.”    “Service users were involved in the assessment process, providing formative feedback on the students' developing service improvement ideas and questions during their assessed poster presentation to a group including a member of staff and their peer group. A short written reflection on the key learning achieved during the module and the post-presentation discussion completed the assessment, with both components marked by staff” | Content analsysis of student summative assessment material, including “a single-slide electronic poster and a single page written summary of post-presentation reflections on learning” | - “The service user experiences that trigger students' choice of service  improvement project;”  - “The project ideas students devised to address identified areas for  improvement in their practice learning context;”  - “Students' perceptions of their key learning from the experience and  its relevance for their qualified practice” | "Students' service improvement IPL can largely be divided into two domains — knowledge development and personal and professional development in relation to themselves (intrapersonal learning), and their relationships with others (interprofessional learning)."    "Interpersonal knowledge gained clearly reflected the taught component of the module “Neglect of a simple task can have a wide negative impact on patients' and relatives' psychological safety”. It also reflected students' experiential learning from undertaking a PDSA cycle: “As a student in an already established team it was essential I used leadership skills to effect change. I needed evidence to convince them of the change I wanted to make”."    "Interprofessional knowledge gained included better role awareness: “I feel I learned a lot from my buddy group … about different professional roles in the health service, the work they're responsible for, difficulties they face, policy drivers and systems they work in, research used and tools they use for assessment, planning and storage of data”.  They also gained knowledge about potential challenges when trying to work interprofessionally to promote patient safety: “I realised the importance of defining professional roles in a team, to prevent role blurring and improve the quality of patient care and safety”. “Overcoming traditional professional differences can be achieved through a joint focus on service user need”.  Knowledge was also gained regarding that the importance of service user involvement in IPL and working: “The thing I will take forward from my learning is the value of the service users' opinion … it has prompted me to modify my professional practice”.  It was clear that the experience had influenced students' personal growth and professional development: “Service improvement involving interprofessional groups and clients  is a complex and crucial subject that I will continue to study and implement throughout my career”,including a perceived increase in their self-confidence as change  agents in an interprofessional context: “I feel better equipped to go into practice and challenge the poor practice of other professionals”.  Some students suggested they had developed an ability to give and receive interprofessional feedback, acknowledging the importance of mutual trust:  “Working with my interprofessional buddy group enabled me to evaluate my own opinions and nursing practice by receiving constructive feedback to learn from positive and negative experiences”.  Many students suggested they now had a more positive interprofessional attitude and had improved their communication skills through their learning experience: “Both my verbal and non verbal communication skills have improved considerably through collaborating with my [interprofessional] buddy group and doing my presentation”.  “I felt pessimistic about both interprofessional working and service improvement but have learned from both”.  “This learning has prompted me to become a ‘collaborative midwife’”.  Not all students had had an entirely positive experience, encountering a range of challenges to the implementation of their projects which affected their learning:  “I would have learned more if I could have actually implemented my plan”.  Other students described persevering to achieve a successful  outcome: “Implementing the project was difficult at first as I came across resistance to change from other staff however once my idea was used once  on the unit, all professionals commented on how useful it was”." | "Service improvement learning in an interprofessional context has positive learning outcomes for health and social care students. Students can identify improvement opportunities that may otherwise go undetected. Engaging positively in interprofessional service improvement learning as a student is an important rehearsal for life as a qualified practitioner. It can help students to develop an ability to challenge unsafe practice elegantly, thereby acting as advocates for the people in their care." |
| Andrew Pinto, Matthew To, Anne Rucchetto, Malik Sharma, Katherine Rouleau; 2018; Canada | Post intervention study without controls | n=17 (11 respondents)    8 health disciplines: health management, chiropractic studies, dietetic studies, family medicine, nurse practitioner, nursing, pharmacy, and social  work | - Interactive lecture sessions with experiential learning (sessions run in collaboration with community partners and individuals with lived experience at locations relevant to the topic for that session);  - Group projects (ranging from literature reviews to policy briefs for government) for presentation at an end-of-year forum | 8 sessions over 1 year | "Course objectives included:  1) promoting interprofessional collaboration;  2) learning about structural barriers and facilitators to attaining “good” health;  3) engaging community-based organizations; and  4) learning appropriate language and skills to advocate for patients”    Interactive lecture sessions themes:  - “Introduction to urban health:”  - “Housing as a determinant of health”  - “Indigenous health in urban environments”  - “Food and income security as determinants of health”  - “Youth and adolescent health in urban environments”  - “Chronic disease management in urban environments”  - “Mental health and addictions in urban environments”  - “Legal status as a determinant of health”    Immersive experiental learning:  “Collaboration with community partners and agencies provided real cases to illustrate patient needs in the urban context. The facilitated visits at community agencies provided students a new perspective on caring for individuals and communities. Community members and front-line workers led these sessions - discussing experiences of stigma, discrimination and how systems failed - to prevent them from lapsing into “safari tourism," where privileged health professionals explore exoticized people in poverty. Consequently, trainees were provided with tools and language for use in advocacy that reflected the input of communities affected. The positioning of people with lived experience as experts was an essential learning element." | Survey with “scale and open-ended comments” | “Participant satisfaction of the course and speaker(s)” | "The average teaching score across 20 different presenters was very high (4.5/5).  Students appreciated the mix of theory and practical examples, with one student noting “Good ideas on how to translate ideas into an effective policy or program.”  Students identified learning from community agencies and people with lived experience as the best aspects of the course. One student commented, “These clients may have different priorities than what we think they need. It is very important to listen to them and see beyond the medical symptoms.”  Students expressed appreciation for new knowledge on SDOH. One student commented: “I learned a lot today that I probably would never have learned if I did not take this elective. It is interesting to see how upstream and downstream thinking can be applied to the discussions today i.e., somehow I feel that the reason why food banks are seen as a solution to hunger is because downstream thinking would identify hunger as the problem and the solution would be to provide food.”  Suggestions for improvement included more support for final projects, greater emphasis on case studies and more clinically-oriented experiential learning." | "The specific health challenges faced in urban settings can benefit from an interprofessional approach informed by the experiences and needs of patient communities. This elective was innovative in engaging students in interprofessional learning on how health and social agencies collaborate to tackle social determinants in urban spaces." |
| Yui Yamaoka, Carisa Wilsie, Elizabeth Bard, Barbara L. Bonner; 2019; United States | Post intervention study without controls | n=508 total participants (179 respondents)    5 health disciplines: psychology, social work, medicine, nursing, public health.    Note: total participants also included ‘law’ and ‘other’ disciplines. | Graduate education course, including:  - Seminars using “video materials, powerpoint presentations and/or discussions with case scenarios.” Speakers included interdisciplinary faculty members and guest speakers from “child protective services, child advocacy groups, domestic violence shelters, and selected agencies.”  - “ITP project to present at the end of year”  - Participation in a “mock trial in front of a judge in a court room”  - “Within discipline practicum/placement”  - Cross discpline practicum/placement: community observations, such as “observations at  juvenile and criminal court, child welfare agencies, Child Advocacy Centers (CAC), a hospital-based Child Protection Team, or an  outpatient treatment program for adult sexual offenders” | Two semesters (9 months) inlcuding:  - weekly 3 hour seminars  - Within discipline placement (6-8 hours/week)  - Cross discipline placement (30 hours/2 semesters) | "The three core objectives of ITP are:  1) to train future leaders who understand the value of each discipline in the prevention of CAN;  2) to provide knowledgeable leaders who are current and lifelong learners in all aspects of the CAN field; and  3) to provide CAN leaders who will, through professional advocacy, education, policy development, research, and service, contribute substantially to the knowledge in the field. Particularly in the field of prevention and treatment for child abuse and neglect, multidisciplinary and inter-agency collaboration is essential due to the complexity of the issues." | Online survey including both quantiative and qualitative questions | “a) the participants’ discipline, b), how their ITP experience related to their  current works, c) any presentations or publications related to child abuse and neglect, d) their current involvement in clinical  practice, research, administration, and prevention, e) knowledge before and after taking the ITP, f) how important was the ITP is for  their long term careers, and g) the percentage of time they were currently involved to CAN-related activities.” | “The participants were asked to check all of the CAN areas in which they were involved. They checked the following areas: child advocacy (43.0% of total responders), education (40.8%), clinical treatment (39.1%), primary prevention (29.6%), and research (27.9%).”  "Table 2 shows how important the ITP lectures and practicum experiences were to the graduates’ professional work. There were no significant differences among discipline groups; however, most of the mean scores exceeded 4 (on a scale of 1–5, with 5 being highest rating) in both didactic lectures and practicums.”  “In terms of didactic lectures, psychology graduates rated the highest score for legal information (m = 4.67, sd = 0.79). Similarly, law students, social workers, and health professionals rated cross discipline-related information higher."  "In terms of practicums, the graduates were asked the importance of the cross-discipline practicums, within-discipline practicums, the mock trial, and the ITP project for their professional careers; there were no significant differences by discipline for the rating."  "There was a significant difference among discipline groups for knowledge about CAN before the ITP (p < 0.001). Social workers had the highest score (m = 7.06, sd = 1.27) and health professionals had the lowest score (m = 4.67, sd = 1.44) on a 1–10 point scale."  "After completing the ITP, a graduate’s knowledge was increased more than 2 points on average, especially for health students (mean increase score = 4.08, sd = 1.83).  The overall satisfaction score was very high (m = 9.31, sd = 0.97).”  “The majority of graduates agreed or strongly agreed that ITP was important for their current job (72.2%) and long-term career (84.7%).”  “In terms of providing leadership in their fields, graduates reported they made 133 presentations to civic or professional groups and published 69 articles since they completed the ITP." | "The ITP trainees have made significant contributions to the field in clinical treatment, research, and child advocacy. Alumni were highly satisfied with their experience and  continue to see the importance of the ITP to their jobs and career." |

Where text is in quotation, this is directly taken from the respective paper in order to prevent the introduction of bias.

*Interpreted by review author (FH) as not explicitly stated within individual studies.
